# Optimal bounds for arithmetic-geometric and Toader means in terms of generalized logarithmic mean

**DOI:** 10.1186/s13660-017-1365-4

**Published:** 2017-05-05

**Authors:** Qing Ding, Tiehong Zhao

**Affiliations:** 1 College of Mathematics and Statistics, Hunan University of Finance and Economics, Changsha, 410205 China; 2 Department of Mathematics, Hangzhou Normal University, Hangzhou, 311121 China

**Keywords:** 26E60, arithmetic-geometric mean, Toader mean, logarithmic mean

## Abstract

In this paper, we find the greatest values $\alpha_{1},\alpha_{2}$ and the smallest values $\beta_{1},\beta_{2}$ such that the double inequalities $L_{\alpha_{1}}(a,b)<\operatorname{AG}(a,b)<L_{\beta_{1}}(a,b)$ and $L_{\alpha_{2}}(a,b)< T(a,b)< L_{\beta_{2}}(a,b)$ hold for all $a, b>0$ with $a\neq b$, where $\operatorname{AG}(a,b)$, $T(a,b)$ and $L_{p}(a,b)$ are the arithmetic-geometric, Toader and generalized logarithmic means of two positive numbers *a* and *b*, respectively.

## Introduction

For $p \in\mathbb{R}$, the *p*th generalized logarithmic mean $L_{p}(a,b)$ [1] of two positive numbers *a* and *b* is defined by

1.1 $$ L_{p}(a,b)= \textstyle\begin{cases} [\frac{b^{p+1}-a^{p+1}}{(p+1)(b-a)} ]^{1/p}, & a\neq b,p\neq-1,p\neq0,\\ \frac{1}{e} (\frac{b^{b}}{a^{a}} )^{1/b-a}, &a\neq b,p=0,\\ \frac{b-a}{\log b-\log a}, & a\neq b, p=-1,\\ a, &a=b. \end{cases} $$

It is well known that $L_{p}(a,b)$ is continuous and strictly increasing with respect to $p\in\mathbb{R}$ for fixed $a,b > 0$ with $a\neq b$. Many remarkable inequalities for the generalized logarithmic mean can be found in the literature [2–17].

The classical arithmetic-geometric mean $\operatorname{AG}(a,b)$ of two positive numbers *a* and *b* is defined by starting with $a_{0}=a,b_{0}=b$ and then iterating

1.2 $$ a_{n+1}=\frac{a_{n}+b_{n}}{2},\qquad b_{n+1}=\sqrt{a_{n}b_{n}} $$

for $n\in\mathbb{N}$ until two sequences $\{a_{n}\}$ and $\{b_{n}\}$ converge to the same number.

The well-known Gauss identity [18] shows that

1.3 $$ \operatorname{AG}(1,r)\mathcal{K}\bigl(\sqrt{1-r^{2}}\bigr)=\frac{\pi}{2} $$

for $r\in(0,1)$, where $\mathcal{K}(r)=\int^{\pi/2}_{0}(1-r^{2}\sin ^{2}t)^{-1/2}\,dt$, $r\in[0,1)$, is the complete elliptic integral of the first kind.

In [19], the Toader mean $T(a,b)$ of two positive numbers *a* and *b* was given by

1.4 $$\begin{aligned} T(a,b) =& \frac{2}{\pi} \int^{\pi/2}_{0}\sqrt{a^{2}\cos^{2} \theta+b^{2}\sin ^{2}\theta}\,d\theta \\ =& \textstyle\begin{cases} \frac{2a\mathcal{E} (\sqrt{1-(b/a)^{2}} )}{\pi}, &a>b,\\ \frac{2b\mathcal{E} (\sqrt{1-(a/b)^{2}} )}{\pi}, &a< b,\\ a,&a=b, \end{cases}\displaystyle \end{aligned}$$

where $\mathcal{E}(r)=\int_{0}^{\pi/2}(1-r^{2}\sin^{2}\theta )^{1/2}\,d\theta$, $r\in[0,1]$ is the complete elliptic integral of the second kind.

Recently, the bounds for the arithmetic-geometric mean $\operatorname{AG}(a,b)$ and Toader mean $T(a,b)$ have attracted the attention of many mathematicians. The double inequality

1.5 $$ L_{-1}(a,b)=L(a,b)< \operatorname{AG}(a,b)< L^{2/3}\bigl(a^{3/2},b^{3/2} \bigr) $$

holds for all $a,b>0$ with $a\neq b$. The left inequality of (1.5) was first proposed by Carlson and Vuorinen [20] and also was proved by different methods in [21–23]. Vamanamurthy and Vuorinen [24] proved that $\operatorname{AG}(a,b)<(\pi/2)L(a,b)$ for all $a,b>0$ with $a\neq b$. The second inequality of (1.5) was proved by Borwein and Borwein [25] and Yang [23].

Vuorinen [26] conjectured that

1.6 $$ M_{3/2}(a,b)< T(a,b) $$

for all $a,b>0$ with $a\neq b$, where $M_{p}(a,b)=[(a^{p}+b^{p})/2]^{1/p}$ ($p\neq0$) and $M_{0}(a,b)=\sqrt{ab}$ is the power mean of order *p*. This conjecture was proved by Qiu and Shen [27] and Barnard et al. [28].

In [29], Alzer and Qiu presented a best possible upper power mean bound for the Toader mean as follows:

1.7 $$ T(a,b)< M_{\log2/\log(\pi/2)}(a,b) $$

for all $a,b>0$ with $a\neq b$.

In [30–32], the authors proved that

1.8 $$\begin{aligned}& \widehat{L}_{0}(a,b)< T(a,b)< \widehat{L}_{1/4}(a,b), \end{aligned}$$

1.9 $$\begin{aligned}& \widehat{S}_{\sqrt{3}/4}(a,b)< T(a,b)< \widehat{S}_{1/2}(a,b) \end{aligned}$$

for all $a,b>0$ with $a\neq b$, where $\widehat {L}_{p}(a,b)=(a^{p+1}+b^{p+1})/(a^{p}+b^{p})$ denotes the *p*th Lehmer mean and $\widehat{S}_{p}(a,b)$ is the generalized Seiffert mean given by $\widehat{S}_{p}(a,b)=p(a-b)/\arctan[2p(a-b)/(a+b)]$ $(0< p\leq1, a\neq b)$, $\widehat{S}_{0}(a,b)=(a+b)/2 (a\neq b)$ and $\widehat{S}_{p}(a,a)=a$.

Very recently, Chu and Wang [33] proved that

1.10 $$\begin{aligned}& S_{p_{1}}(a,b)< \operatorname{AG}(a,b)< S_{q_{1}}(a,b), \end{aligned}$$

1.11 $$\begin{aligned}& S_{p_{2}}(a,b)< T(a,b)< S_{q_{2}}(a,b) \end{aligned}$$

for all $a,b>0$ with $a\neq b$ if and only if $p_{1}\leq1/2,q_{1}\geq1$ and $p_{2}\leq1,q_{2}\geq3/2$. Here the *p*th Gini mean of two positive numbers *a* and *b* is defined by

1.12 $$ S_{p}(a,b)= \textstyle\begin{cases} (\frac{a^{p-1}+b^{p-1}}{a+b} )^{1/(p-2)}, &p\neq2,\\ (a^{a}b^{b})^{1/(a+b)}, &p=2. \end{cases} $$

The main purpose of this paper is to find the greatest values $\alpha _{1},\alpha_{2}$ and the smallest values $\beta_{1},\beta_{2}$ such that the double inequalities $L_{\alpha_{1}}(a,b)<\operatorname{AG}(a,b)<L_{\beta_{1}}(a,b)$ and $L_{\alpha_{2}}(a,b)< T(a,b)< L_{\beta_{2}}(a,b)$ hold for all $a, b>0$ with $a\neq b$ and give some new bounds for the complete elliptic integrals.

## Basic knowledge and lemmas

In order to prove our main results, we need several formulas and lemmas, which we present in this section.

For $r\in(0, 1)$ and $r'=\sqrt{1-r^{2}}$, the well-known complete elliptic integrals of the first and second kinds are defined by

$$ \textstyle\begin{cases} \mathcal{K}=\mathcal{K}(r)=\int^{\pi/2}_{0}(1-r^{2}\sin^{2}\theta )^{-1/2}\,d\theta,\\ \mathcal{K}'=\mathcal{K}'(r)=\mathcal{K}(r'),\\ \mathcal{K}(0)=\pi/2,\qquad\mathcal{K}(1)=+\infty, \end{cases} $$

and

$$ \textstyle\begin{cases} \mathcal{E}=\mathcal{E}(r)=\int^{\pi/2}_{0}(1-r^{2}\sin^{2}\theta )^{1/2}\,d\theta,\\ \mathcal{E}'=\mathcal{E}'(r)=\mathcal{E}(r')\\ \mathcal{E}(0)=\pi/2,\qquad\mathcal{E}(1)=1, \end{cases} $$

respectively, and the following formulas were presented in [18], Appendix E, pp.474-475:

2.1 $$\begin{aligned} \begin{aligned} & \frac{d\mathcal{K}}{dr}=\frac{\mathcal{E}-r^{\prime 2}\mathcal {K}}{rr^{\prime 2} },\qquad \frac{d\mathcal{E}}{dr}=\frac{\mathcal {E}-\mathcal{K}}{r}, \\ & \frac{d(\mathcal{E}-r^{\prime 2}\mathcal{K})}{dr}=r\mathcal{K},\qquad \frac {d(\mathcal{K}-\mathcal{E})}{dr}=\frac{r\mathcal{E}}{r^{\prime 2}}, \end{aligned} \end{aligned}$$

2.2 $$\begin{aligned} & \mathcal{E} \biggl(\frac{2\sqrt{r}}{1+r} \biggr)=\frac{2\mathcal {E}-r^{\prime 2}\mathcal{K}}{1+r}. \end{aligned}$$

In what follows, four special values $\mathcal{E}(\sqrt {2}/2),\mathcal{K}(\sqrt{2}/2)$ and $\mathcal{E}(0.9),\mathcal {K}(0.9)$ will be used. By numerical computations, these are given by

2.3 $$\begin{aligned}& \mathcal{E}(\sqrt{2}/2) =1.35064\cdots,\qquad\mathcal{K}(\sqrt {2}/2)=1.85407 \cdots, \end{aligned}$$

2.4 $$\begin{aligned}& \mathcal{E}(0.9)=1.1717\cdots,\qquad\mathcal{K}(0.9)=2.28055\cdots. \end{aligned}$$

### Lemma 2.1

See [18], Theorem 1.25

*For* $-\infty< a < b <\infty$, *let* $f,g:[a, b]\rightarrow\mathbb{R}$ *be continuous on* $[a, b]$ *and be differentiable on* $(a, b)$, *let* $g'(x)\neq0$ *on* $(a, b)$. *If* $f'(x)/g'(x)$ *is increasing* (*decreasing*) *on* $(a, b)$, *then so are*

$$ \frac{f(x)-f(a)}{g(x)-g(a)}\quad\textit{and}\quad\frac {f(x)-f(b)}{g(x)-g(b)}. $$

*If* $f '(x)/g'(x)$ *is strictly monotone*, *then the monotonicity in the conclusion is also strict*.

### Lemma 2.2

(1) *The function* $r\rightarrow(\mathcal{E}-r^{\prime 2}\mathcal{K})/r^{2}$ *is strictly increasing from* $(0, 1)$ *onto* $(\pi/4, 1)$;

(2) *The function* $r\rightarrow2\mathcal{E}-r^{\prime 2}\mathcal{K}$ *is increasing and log*-*convex from* $(0,1)$ *onto* $(\pi/2,2)$;

(3) *The function* $\mathcal{K}/\log(e^{2}/r')$ *is strictly increasing from* $(0,1)$ *onto* $(\pi/4, 1)$;

(4) *The function* $(\mathcal{K}-\mathcal{E})/r^{2}$ *is strictly increasing on* $(0,1)$; *in particular*, $\mathcal{K}-\mathcal{E}>(\pi /4)r^{2}$ *for all* $r\in(0,1)$.

### Proof

Parts (1) and (2) follow from [18], Theorem 3.21(1) and Exercise 3.43(13). □

### Lemma 2.3

*The equation*

$$(1+p)^{1/p}=\frac{\pi}{2} $$

*has a unique solution* $p=p_{0}=3.15295\cdots$ .

### Proof

Let

$$ \varphi(p)= \textstyle\begin{cases} (1+p)^{1/p}-\pi/2,& p\in(-1,0)\cup(0,+\infty),\\ e-\pi/2,&p=0. \end{cases} $$

It is easy to verify that the function *φ* is continuous and strictly decreasing from $(-1,+\infty)$ onto $(1,+\infty)$. Therefore, Lemma 2.3 easily follows from the continuity and monotonicity of *φ* together with the facts that $\varphi(3.15295)=6.14999\times10^{-7}$ and $\varphi (3.15296)=-4.35155\times10^{-7}$. □

### Lemma 2.4

*The function*

$$f(r)=\frac{2(2\mathcal{E}-r^{\prime 2}\mathcal{K})/\pi-1-r^{2}/4}{r^{4}} $$

*is strictly increasing from* $(0,1)$ *onto* $(1/64,4/\pi-5/4)$.

### Proof

Let $f_{1}(r)=2(2\mathcal{E}-r^{\prime 2}\mathcal{K})/\pi-1-r^{2}/4$ and $\widehat{f}_{1}(r)=r^{4}$, then $f_{1}(0)=\widehat{f}_{1}(0)=0$ and $f(r)=f_{1}(r)/\widehat{f}_{1}(r)$.

A simple calculation yields

2.5 $$\begin{aligned}& \frac{f'_{1}(r)}{\widehat{f}'_{1}(r)}=\frac{4(\mathcal{E}-r^{\prime 2}\mathcal {K})-\pi r^{2}}{8\pi r^{4}}\triangleq\frac{f_{2}(r)}{\widehat{f}_{2}(r)}, \end{aligned}$$

2.6 $$\begin{aligned}& f_{2}(0)=\widehat{f}_{2}(0), \end{aligned}$$

2.7 $$\begin{aligned}& \frac{f'_{2}(r)}{\widehat{f}'_{2}(r)}=\frac{2\mathcal{K}-\pi}{16\pi r^{2}}\triangleq\frac{f_{3}(r)}{\widehat{f}_{3}(r)}, \end{aligned}$$

2.8 $$\begin{aligned}& f_{3}(0)=\widehat{f}_{3}(0), \end{aligned}$$

2.9 $$\begin{aligned}& \frac{f'_{3}(r)}{\widehat{f}'_{3}(r)}=\frac{1}{16\pi}\cdot\frac {\mathcal{E}-r^{\prime 2}\mathcal{K}}{r^{2}}\cdot \frac{1}{r^{\prime 2}}. \end{aligned}$$

Following from Lemma 2.2(1) and (2.9) together with the monotonicity of $1/r^{\prime 2}$, we clearly see that $f'_{3}(r)/\widehat{f}'_{3}(r)$ is strictly increasing on $(0,1)$. Equations (2.5)-(2.8) and Lemma 2.1 lead to the conclusion that $f(r)$ is strictly increasing on $(0,1)$.

Therefore, Lemma 2.4 follows from the monotonicity of $f(r)$ together with the facts that $f(0^{+})=1/64$ and $f(1^{-})=4/\pi-5/4$. □

The following double inequalities can be obtained from Lemma 2.4 immediately.

### Corollary 2.5

*Inequalities*

$$1+\frac{r^{2}}{4}+\frac{r^{4}}{64}< \frac{2}{\pi}\bigl(2\mathcal {E}-r^{\prime 2}\mathcal{K}\bigr)< 1+\frac{r^{2}}{4}+ \biggl( \frac{4}{\pi}-\frac {5}{4} \biggr)r^{4} $$

*hold for* $0< r<1$.

### Lemma 2.6

*The inequality*

2.10 $$ \biggl[\frac{(1+r)^{7/2}-(1-r)^{7/2}}{7r} \biggr]^{2/5}< 1+\frac{r^{2}}{4} $$

*holds for* $0< r<1$.

### Proof

In order to prove inequality (2.10), it suffices to prove that

2.11 $$\begin{aligned} g(r) =& \bigl[(1+r)^{7/2}-(1-r)^{7/2} \bigr]^{2}-49r^{2} \biggl(1+\frac{r^{2}}{4} \biggr)^{5} \\ =& (1+r)^{7}+(1-r)^{7}-49r^{2} \biggl(1+ \frac{r^{2}}{4} \biggr)^{5}-2\bigl(1-r^{2} \bigr)^{7/2} \\ =& g_{1}(r)-g_{2}(r)< 0 \end{aligned}$$

for $0< r<1$, where

$$\begin{aligned}& g_{1}(r)=(1+r)^{7}+(1-r)^{7}-49r^{2} \biggl(1+\frac{r^{2}}{4} \biggr)^{5}, \\& g_{2}(r)=2\bigl(1-r^{2}\bigr)^{7/2}. \end{aligned}$$

Observe that

2.12 $$\begin{aligned}& g'_{1}(r)=-\frac{7r}{256} \bigl[32 \bigl(4-5r^{2}\bigr)^{2}+2{,}848r^{4} + 2{,}240r^{6} + 350 r^{8} + 21 r^{10} \bigr]< 0, \end{aligned}$$

2.13 $$\begin{aligned}& g_{1}(0.56)=0.0755\cdots>0,\qquad g_{1}(0.57)=-0.00966 \cdots< 0, \end{aligned}$$

we conclude, from (2.12) and (2.13), that there exists $r_{0}\in (0.56,0.57)$ such that $g_{1}(r)>0$ for $r\in(0,r_{0})$ and $g_{1}(r)<0$ for $r\in(r_{0},1)$.

In order to prove (2.11), we divide it into two cases.

*Case* A $r\in[r_{0},1)$. In this case, we clearly see that $g_{1}(r)\leq0$ and $g_{2}(r)>0$. This implies that $g(r)=g_{1}(r)-g_{2}(r)<0$.

*Case* B $r\in(0,r_{0})$. In this case, $g_{1}(r)>0$. Let $g_{3}(r)=2-7r^{2}+\frac{35}{4}r^{4}-6r^{6}$, the difference between $g_{1}(r)$ and $g_{3}(r)$ yields

2.14 $$ g_{1}(r)-g_{3}(r)=-\frac{r^{6}(10{,}880 + 7{,}840 r^{2} + 980 r^{4} + 49 r^{6})}{1{,}024}< 0. $$

We know from (2.14) that $g_{3}(r)>g_{1}(r)>0$. Moreover,

$$ g_{3}^{2}(r)-g_{2}^{2}(r)=- \frac{r^{6}}{16}\bigl[\bigl(2-4r^{2}\bigr) \bigl(4+r^{2} \bigr)+r^{2}\bigr] \biggl[16 \biggl(r^{2}-\frac{5}{8} \biggr)+\frac{27}{4} \biggr]< 0, $$

this in conjunction with $g_{3}(r)>0$ implies that

2.15 $$ g_{3}(r)-g_{2}(r)< 0. $$

Therefore, we clearly see that $g(r)=[g_{1}(r)-g_{3}(r)]+[g_{3}(r)-g_{2}(r)]<0$ from (2.14) and (2.15). □

### Lemma 2.7

*Let* $\eta(r)=[(1+r)^{p_{0}+1}-(1-r)^{p_{0}+1}]/r$ *and* $\omega (r)=[(1-r)^{p_{0}}(1+p_{0}r)-(1+r)^{p_{0}}(1-p_{0}r)]/r^{2}$, *then the functions* $\eta(r)$ *and* $\omega(r)$ *both are strictly increasing on* $(0,1)$.

### Proof

We assume that

$$\begin{aligned}& \eta_{1}(r)=(1+r)^{p_{0}+1}-(1-r)^{p_{0}+1},\qquad \eta_{2}(r)=r, \\& \omega_{1}(r)=(1-r)^{p_{0}}(1+p_{0}r)-(1+r)^{p_{0}}(1-p_{0}r), \qquad\omega_{2}(r)=r^{2}, \end{aligned}$$

then $\eta(r)=\eta_{1}(r)/\eta_{2}(r)$ and $\omega(r)=\omega _{1}(r)/\omega_{2}(r)$.

A simple calculation yields

2.16 $$\begin{aligned}& \eta_{1}(0)=\eta_{2}(0)=\omega_{1}(0)= \omega_{2}(0)=0, \end{aligned}$$

2.17 $$\begin{aligned}& \frac{\eta'_{1}(r)}{\eta'_{2}(r)}=(1+p_{0})\bigl[(1+r)^{p_{0}}-(1-r)^{p_{0}} \bigr], \end{aligned}$$

2.18 $$\begin{aligned}& \frac{\omega'_{1}(r)}{\omega'_{2}(r)}=\frac {p_{0}(p_{0}+1)[(1+r)^{p_{0}-1}-(1-r)^{p_{0}-1}]}{2}. \end{aligned}$$

Lemma 2.1 and (2.16)-(2.18) lead to the conclusion that $\eta(r)$ and $\omega(r)$ are strictly increasing on $(0,1)$. □

### Lemma 2.8

*Let*

$$ \phi_{p}(r)=\frac{2}{\pi} \bigl(2\mathcal{E}-r^{\prime 2} \mathcal{K} \bigr)- \biggl[\frac{(1+r)^{p+1}-(1-r)^{p+1}}{2(p+1)r} \biggr]^{1/p}, $$

*then* $\phi_{p}(r)>0$ *for* $0< r<1$ *if and only if* $p\leq5/2$; $\phi _{p}(r)<0$ *for* $0< r<1$ *if and only if* $p\geq p_{0}$.

### Proof

It is well known that $L_{p}(a,b)$ is strictly increasing with respect to $p\in\mathbb{R}$ for fixed $a,b>0$ with $a\neq b$, then $\phi_{p}(r)$ is strictly decreasing with respect to $p\in\mathbb{R}$. In order to prove Lemma 2.8, we divide it into three cases.

*Case* 1 $p=5/2$.

From Corollary 2.5 and Lemma 2.6, we clearly see that

$$\begin{aligned} \phi_{5/2}(r) =& \frac{2}{\pi} \bigl(2\mathcal{E}-r^{\prime 2} \mathcal {K} \bigr)- \biggl[\frac{(1+r)^{7/2}-(1-r)^{7/2}}{7r} \biggr]^{2/5} \\ >& 1+\frac{r^{2}}{4}+\frac{r^{4}}{64}- \biggl[\frac {(1+r)^{7/2}-(1-r)^{7/2}}{7r} \biggr]^{2/5} \\ >& \frac{r^{4}}{64}>0 \end{aligned}$$

for $0< r<1$.

*Case* 2 $p=p_{0}$.

We divide it into two subcases.

*Subcase* A $\phi_{p_{0}}(r)<0$ for $r\in(0,0.9)$.

Since $\phi_{p}(r)$ is strictly decreasing with respect to $p\in\mathbb {R}$, we clearly see that $\phi_{p_{0}}(r)<\phi_{3}(r)$. It suffices to prove that $\phi_{3}(r)<0$ for $r\in(0,0.9)$.

For $r\in(0,\sqrt{2}/2]$, it follows from Corollary 2.5 that

$$\begin{aligned} \phi_{3}(r) =& \frac{2}{\pi} \bigl(2\mathcal{E}-r^{\prime 2} \mathcal{K} \bigr)- \biggl[\frac{(1+r)^{4}-(1-r)^{4}}{8r} \biggr]^{1/3} \\ < & 1+\frac{r^{2}}{4}+ \biggl(\frac{4}{\pi}-\frac{5}{4} \biggr)r^{4}- \biggl(1+\frac{r^{2}}{3}-\frac{r^{4}}{9} \biggr)^{3} \\ = & -\frac{r^{2}}{12} \biggl[1- \biggl(\frac{48}{\pi}-\frac{41}{3} \biggr)r^{2} \biggr] \\ \leq & -\frac{r^{2}}{12} \biggl[1-\frac{1}{2} \biggl(\frac{48}{\pi }- \frac{41}{3} \biggr) \biggr] \\ =& -\frac{(47\pi-144)r^{2}}{72\pi}< 0, \end{aligned}$$

where the first inequality easily follows from

$$ \frac{(1+r)^{4}-(1-r)^{4}}{8r}- \biggl(1+\frac{r^{2}}{3}-\frac {r^{4}}{9} \biggr)^{3}=\frac{r^{6}}{729}\bigl[126+9\bigl(1-r^{4} \bigr)+r^{6}\bigr]>0. $$

For $r\in(\sqrt{2}/2,0.9)$, taking the derivative of $\phi_{3}(r)$ yields

2.19 $$ \phi'_{3}(r)=\frac{2(\mathcal{E}-r^{\prime 2}\mathcal{K})}{\pi r}-\frac {2r}{3(1+r^{2})^{2/3}}= \mu_{1}(r)+\mu_{2}(r), $$

where

$$ \mu_{1}(r)=\frac{2(\mathcal{E}-r^{\prime 2}\mathcal{K})}{\pi r}-\frac {r}{2},\qquad \mu_{2}(r)=\frac{r}{2}-\frac{2r}{3(1+r^{2})^{2/3}}. $$

From Lemma 2.2(4), we clearly see that

2.20 $$ \frac{d\mu_{1}(r)}{dr}=\frac{2}{\pi r^{2}} \biggl(\mathcal{K}-\mathcal {E}- \frac{\pi}{4}r^{2} \biggr)>0 $$

for $r\in(0,1)$ and

2.21 $$\begin{aligned} \frac{d\mu_{2}(r)}{dr} =& \frac {9(1+r^{2})^{5/3}-12+4r^{2}}{18(1+r^{2})^{5/3}}> \frac {9[1+(5/3)r^{2}]-12+4r^{2}}{18(1+r^{2})^{5/3}} \\ =& \frac{19r^{2}-3}{18(1+r^{2})^{5/3}}>0 \end{aligned}$$

for $r\in(\sqrt{2}/2,0.9)$. Equations (2.19)-(2.21) lead to the conclusion that $\phi'_{3}(r)$ is strictly increasing on $(\sqrt {2}/2,0.9)$. This in conjunction with (2.3) implies that

2.22 $$ \phi'_{3}(r)>\phi'_{3}( \sqrt{2}/2)=0.02163\cdots>0 $$

for $r\in(\sqrt{2}/2,9/10)$. Therefore, from (2.22) we clearly see that $\phi_{3}(r)$ is strictly increasing on $(\sqrt{2}/2,0.9)$. This in conjunction with (2.4) yields $\phi_{3}(r)<\phi _{3}(0.9)=-0.002687\cdots<0$ for $r\in(\sqrt{2}/2,0.9)$.

*Subcase* B $\phi_{p_{0}}(r)<0$ for $r\in[0.9,1)$.

For $0.9\leq r<1$, taking the derivation of $\phi_{p_{0}}(r)$ yields

2.23 $$ \phi'_{p_{0}}(r)=\frac{2(\mathcal{E}-r^{\prime 2}\mathcal{K})}{\pi r}-\frac {\omega(r)}{p_{0}(p_{0}+1)2^{1/p_{0}}\eta(r)^{1-1/p_{0}}}, $$

where $\omega(r)$ and $\eta(r)$ are defined as in Lemma 2.7. From Lemma 2.2(1), we clearly see that $(\mathcal{E}-r^{\prime 2}\mathcal{K})/r$ is strictly increasing on $(0,1)$. Lemma 2.7 and (2.4), (2.20) lead to the conclusion that

$$\begin{aligned} \phi'_{p_{0}}(r) \geq & \frac{2[\mathcal{E}(0.9)-(1-0.9^{2})\mathcal {K}(0.9)]}{0.9\pi}-\frac{\omega(1)}{p_{0}(p_{0}+1)2^{1/p_{0}}\eta (0.9)^{1-1/p_{0}}} \\ =& 0.522306\cdots-0.46787\cdots=0.054436\cdots>0 \end{aligned}$$

for $0.9\leq r<1$.

Therefore, it follows from the monotonicity of $\phi'_{p_{0}}(r)$ on $(9/10,1)$ that $\phi_{p_{0}}(r)<\phi_{p_{0}}(1)=2 [2/\pi -1/(1+p_{0})^{1/p_{0}} ]=0$ for $0< r<1$.

*Case* 3 $5/2< p< p_{0}$.

Taking the Taylor series of $\phi_{p}(r)$ at $r=0$ yields

2.24 $$ \phi_{p}(r)= \biggl(\frac{5}{12}-\frac{p}{6} \biggr)r^{2}+\frac{(149 - 144 p + 24 p^{2} + 16 p^{3}) r^{4}}{2{,}880}+o\bigl(r^{4}\bigr). $$

From (2.24) we clearly see that there exists a sufficiently small $\delta_{1}>0$ such that $\phi_{p}(r)<0$ for $r\in(0,\delta_{1})$ if $p>5/2$. If $p< p_{0}$, then $\phi_{p}(1)=2[2/\pi-1/(1+p)^{1/p}]>0$. By the continuity of $\phi_{p}(r)$ with respect to *r*, there exists a sufficiently small $\delta_{2}>0$ such that $\phi_{p}(r)>0$ for $r\in (\delta_{2},1)$. □

## Main results

### Theorem 3.1

*Inequality* $L_{-1}(a,b)<\operatorname{AG}(a,b)<L_{-1/2}(a,b)$ *holds for all* $a, b>0$ *with* $a\neq b$, *where* $L_{-1}(a,b)$ *and* $L_{-1/2}(a,b)$ *are the best possible lower and upper generalized logarithmic mean bounds for the arithmetic*-*geometric mean* $\operatorname{AG}(a,b)$, *respectively*.

### Proof

Firstly, from (1.5) we clearly see that $L_{-1}(a,b)<\operatorname{AG}(a,b)$ for all $a, b>0$ with $a\neq b$.

Next, we prove that $\operatorname{AG}(a,b)< L_{-1/2}(a,b)$ for all $a, b>0$ with $a\neq b$. Since $\operatorname{AG}(a,b)$ and $L_{p}(a,b)$ are symmetric and homogeneous of degree 1, without loss of generality, it suffices to give an assumption that $a=1 > b$. Let $t=b\in(0,1)$, $r=(1-t)/(1+t)$, then (1.1) and (1.3) lead to

3.1 $$\begin{aligned} \operatorname{AG}(a,b)-L_{-1/2}(a,b) =& \frac{\pi}{2\mathcal{K}(\sqrt{1-t^{2}})}- \biggl[\frac{1-t}{2(1-\sqrt{t})} \biggr]^{2} \\ =& \frac{1}{1+r} \biggl[\frac{\pi}{2\mathcal{K}}- \biggl(\frac {r}{\sqrt{1+r}-\sqrt{1-r}} \biggr)^{2} \biggr] \\ =& \frac{h(r)}{2(1+r)\mathcal{K}(r)}, \end{aligned}$$

where

$$ h(r)=\pi-\bigl(1+r'\bigr)\mathcal{K}(r). $$

We can rewrite $h(r)$ as

3.2 $$ h(r)=\pi-\lambda\bigl(r'\bigr)\cdot\frac{\mathcal{K}}{\log(e^{2}/r'2)}, $$

where $\lambda(r')=(1+r')\log(e^{2}/r')$.

A simple calculation yields

3.3 $$\begin{aligned}& \lambda'\bigl(r'\bigr)=1-\frac{1}{r'}-\log r', \end{aligned}$$

3.4 $$\begin{aligned}& \lambda'(1)=0, \end{aligned}$$

3.5 $$\begin{aligned}& \lambda''\bigl(r'\bigr)= \frac{1-r'}{r'}>0. \end{aligned}$$

Equations (3.3)-(3.5) lead to the conclusion that $\lambda(r')$ is strictly decreasing on $(0,1)$ with respect to $r'$. Moreover, the function $r'=\sqrt{1-r^{2}}$ is strictly decreasing on $(0,1)$. Hence the function $\lambda(r')$ is strictly increasing on $(0,1)$ with respect to *r*. It follows from (3.2) and Lemma 2.2(3) that $h(r)$ is strictly decreasing on $(0,1)$. This implies that $h(r)<0$ for $0< r<1$ together with $h(0)=0$.

Therefore, $\operatorname{AG}(a,b)< L_{-1/2}(a,b)$ for all $a, b>0$ with $a\neq b$ follows from (3.1) and $h(r)<0$.

Finally, we prove that $L_{-1}(a,b)$ and $L_{-1/2}(a,b)$ are the best possible lower and upper generalized logarithmic mean bounds for the arithmetic-geometric mean $\operatorname{AG}(a,b)$.

For any $0<\varepsilon<1/2$ and $0< x<1$, it follows from (1.1) and (1.3) that

3.6 $$\begin{aligned} \lim _{x\rightarrow0}\bigl[\operatorname{AG}(1,x)-L_{-1+\varepsilon}(1,x)\bigr] =& \lim _{x\rightarrow0} \biggl\{ \frac{\pi}{2\mathcal{K}(\sqrt {1-x^{2}})}- \biggl[\frac{1-x^{\varepsilon}}{\varepsilon(1-x)} \biggr]^{\frac{1}{\varepsilon-1}} \biggr\} \\ =& -\varepsilon^{\frac{1}{1-\varepsilon}} \end{aligned}$$

and making use of the Taylor expansion as $x\rightarrow0$, one has

3.7 $$\begin{aligned}& \operatorname{AG}(1,1-x)-L_{-1/2-\varepsilon}(1,1-x) \\ & \quad=\frac{\pi}{2\mathcal{K}(\sqrt{2x-x^{2}})}- \biggl[\frac {1-(1-x)^{1/2-\varepsilon}}{(1/2-\varepsilon)x} \biggr]^{-\frac {2}{1+2\varepsilon}} \\ & \quad= \biggl[1-\frac{x}{2}-\frac{x^{2}}{16}+o \bigl(x^{2}\bigr) \biggr]- \biggl[1-\frac {x}{2}- \frac{3+2\varepsilon}{48}x^{2}+o\bigl(x^{2}\bigr) \biggr] \\ & \quad=\frac{\varepsilon}{24}x^{2}+o\bigl(x^{2}\bigr). \end{aligned}$$

Equations (3.6) and (3.7) imply that for any $0<\varepsilon<1/2$ there exist $\delta_{1}=\delta_{1}(\varepsilon)\in(0,1)$ and $\delta _{2}=\delta_{2}(\varepsilon)\in(0,1)$ such that $\operatorname{AG}(1,x)< L_{-1+\varepsilon}(1,x)$ for $x\in(0,\delta_{1})$ and $\operatorname{AG}(1,1-x)>L_{-1/2-\varepsilon}(1,1-x)$ for $x\in(0,\delta_{2})$. □

### Theorem 3.2

*Inequality* $L_{5/2}(a,b)< T(a,b)< L_{p_{0}}(a,b)$ *holds for all* $a, b>0$ *with* $a\neq b$, *where* $p_{0}$ *is defined as in Lemma* 2.3 *and* $L_{5/2}(a,b), L_{p_{0}}(a,b)$ *are the best possible lower and upper generalized logarithmic mean bounds for the Toader mean* $T(a,b)$, *respectively*.

### Proof

From (1.1) and (1.4) we clearly see that both $T(a,b)$ and $L_{p}(a,b)$ are symmetric and homogeneous of degree 1. Without loss of generality, we assume that $a=1 > b$. Let $t=b\in(0,1)$, $r=(1-t)/(1+t)$, then from (1.1) and (1.4) together with (2.2) we have

3.8 $$\begin{aligned} T(a,b)-L_{p}(a,b) =& \frac{2}{\pi} \mathcal{E}\bigl(\sqrt{1-t^{2}}\bigr)- \biggl[\frac{1-t^{p+1}}{(p+1)(1-t)} \biggr]^{1/p} \\ =& \frac{2}{\pi}\mathcal{E} \biggl(\frac{2\sqrt{r}}{1+r} \biggr)- \frac{1}{1+r} \biggl[\frac{(1+r)^{p+1}-(1-r)^{p+1}}{2(p+1)r} \biggr]^{1/p} \\ =& \frac{1}{1+r} \biggl[\frac{2}{\pi} \bigl(2\mathcal{E}-r^{\prime 2} \mathcal {K} \bigr)- \biggl(\frac{(1+r)^{p+1}-(1-r)^{p+1}}{2(p+1)r} \biggr)^{1/p} \biggr] \\ =& \frac{\phi_{p}(r)}{(1+r)}, \end{aligned}$$

where $\phi_{p}(r)$ is defined as in Lemma 2.8.

Therefore, Theorem 3.2 follows from (3.8) and Lemma 2.8. □

## Corollaries and remarks

From Theorem 3.1 we get a lower bound for the complete elliptic integral of the first kind $\mathcal{K}(r)$ as follows.

### Corollary 4.1

*Inequality*

4.1 $$ \mathcal{K}(r)>\frac{2\pi[1+\sqrt{1-r^{2}}-2(1-r^{2})^{1/4}]}{(1-\sqrt {1-r^{2}})^{2}} $$

*holds for all* $r\in(0,1)$.

### Remark 4.1

We define $H(r)=2\pi[1+\sqrt{1-r^{2}}-2(1-r^{2})^{1/4}]/ (1-\sqrt{1-r^{2}} )^{2}$. Computational and numerical experiments show that the lower bound in (4.1) can be regarded as an approximation of $\mathcal{K}(r)$ for some $r\in(0,1)$, refer to Table 1 for numerical values.

**Table 1:** **Comparison of** $\pmb{\mathcal{K}(r)}$ **with** $\pmb{H(r)}$ **for some** $\pmb{r\in(0,1)}$

***r***	$\boldsymbol{\mathcal{K}(r)}$	***H*** **(** ***r*** **)**
0.1	1.5747455615⋯	1.5747455614⋯
0.2	1.5868678474⋯	1.5868678471⋯
0.3	1.608048619⋯	1.608048612⋯
0.4	1.63999986⋯	1.63999977⋯
0.5	1.68575035⋯	1.68574965⋯
0.6	1.75075380⋯	1.75074958⋯
0.7	1.84569400⋯	1.84567106⋯
0.8	1.99530278⋯	1.99517293⋯

Theorem 3.2 enables us to give new bounds for the complete elliptic integrals of the second kind $\mathcal{E}(r)$.

### Corollary 4.2

*Inequality*

4.2 $$ \frac{\pi}{2} \biggl[\frac{2(1-(1-r^{2})^{7/4})}{7(1-\sqrt {1-r^{2}})} \biggr]^{2/5}< \mathcal{E}(r)< \frac{\pi}{2} \biggl[\frac {1-(1-r^{2})^{(p_{0}+1)/2}}{(p_{0}+1)(1-\sqrt{1-r^{2}})} \biggr]^{1/p_{0}} $$

*holds for all* $r\in(0,1)$, *where* $p_{0}=3.15295\cdots$ *is defined as in Lemma* 2.3.
